# Scaling-Up Access to Family Planning May Improve Linear Growth and Child Development in Low and Middle Income Countries

**DOI:** 10.1371/journal.pone.0102391

**Published:** 2014-07-14

**Authors:** Günther Fink, Christopher R. Sudfeld, Goodarz Danaei, Majid Ezzati, Wafaie W. Fawzi

**Affiliations:** 1 Harvard School of Public Health, Boston, Massachusetts, United States of America; 2 MRC-PHE Centre for Environment and Health, Departments of Epidemiology and Biostatistics, Imperial College London, London, United Kingdom; University of Southampton, United Kingdom

## Abstract

**Background:**

A large literature has indicated a robust association between birth spacing and child survival, but evidence on the association of birth timing with physical growth in low and middle income countries (LMICs) remains limited.

**Methods and Results:**

Data from 153 cross-sectional Demographic and Health Surveys (DHS) across 61 LMICs conducted between 1990 and 2011 were combined to assess the association of birth timing with child stunting (height-for-age z-score <−2). A total of 623,789 children of birth order 1–5 contributed to the maternal age analysis, while the birth spacing dataset consisted of 584,226 children of birth order 2 and higher. Compared to 27–34 year old mothers, maternal age under 18 years was associated with a relative stunting risk of 1.35 (95% CI: 1.29–1.40) for firstborn children, whereas the relative risk was 1.24 (95% CI: 1.19–1.29) for mothers aged 18–19 years. The association of young maternal age with stunting was significantly greater for urban residents and those in the top 50% of household wealth. Birth intervals less than 12 months and 12–23 months had relative risks for stunting of 1.09 (95% CI: 1.06–1.12) and 1.06 (95% CI: 1.05–1.06) as compared to a 24–35 month inter-pregnancy interval, respectively. The strength of both teenage pregnancy and short birth interval associations showed substantial variation across WHO region. We estimate that 8.6% (6.9–10.3%) of stunted cases in the South Asian DHS sample would have been averted by jointly eliminating teen pregnancies and birth intervals less than 24 months, while only 3.6% (1.5–5.7%) of stunting cases would have prevented in the Middle East and North Africa sample.

**Conclusions:**

Postponing the age of first birth and increasing inter-pregnancy intervals has the potential to significantly reduce the prevalence of stunting and improve child development in LMICs.

## Introduction

Approximately 16 million teenagers (under 20 years of age) give birth each year worldwide, of which more than 90% reside in low and middle income countries (LMICs) [1]. Childbirth at an early age and short birth spacing have been shown to be associated with increased risk of birth complications [2], child mortality [3]–[5], and physical growth restrictions [4], [6]–[10]. Studies primarily conducted in high income settings have also found young maternal age to be associated with poor cognitive and behavioral outcomes for children [11], [12].

Approximately one-third of children under age 5 in LMICs, or about 314 million children, are currently affected by linear growth restriction or stunting [13]. Stunting has long been recognized as a principal risk factor for child morbidity and mortality, but more recent work has also shown consistent associations with cognitive deficits and underachievement in school [14], [15] and lower adult earnings [14], [16], [17].

While previous studies have documented associations between birth timing and child physical growth [4], [18], [19], relatively little is known about the relative magnitude of these associations across socioeconomic and cultural settings. Given the complex biological, social, and behavioral mechanisms underlying birth timing [20]–[22], there variations across regions as well as within countries are potentially large [23]. In this study, we use the most comprehensive global dataset with birth timing available to date in order to update risk estimates of young maternal age and short birth spacing with child linear growth, examine differences in the associations across geographic and socioeconomic strata, and quantify the potential impact of eliminating high risk birth timing on the prevalence of child stunting in LMICs.

## Methods

### Data Sources

The dataset utilized for this study was pooled from 153 cross-sectional Demographic and Health Surveys (DHS) conducted 1990 to 2011 in 61 low and middle income countries. DHS are nationally representative surveys of households that collect a wide-range of data with emphasis on maternal and child health indicators. The 61 DHS countries (shown in Table S1 and Figure S4 in File S1) included in this dataset cover 83% of the total population residing low-income countries and 48% of the population of middle-income countries as classified by the World Bank in 2010 [24].

### Study Population

In total 768,504 children aged 6–59 months were included in the 153 DHS which included child anthropometric measurements. A total of 17,962 children (1.1%) were excluded due to implausible height-for-age z-scores (HAZ) (< -6SD or >6SD), and an additional 138 children (0.1%) were excluded due to missing covariate information. The maternal age analysis was restricted to 623,789 children of birth order 1–5 due to the implausibility of having more than five children during the teenage years, while the birth interval dataset included all 584,226 children of birth order 2 and higher.

### Exposures, Covariates, and Outcomes

Maternal age and birth spacing was assessed by self-report of the mother. Given that exact dates of conception are not available within the DHS dataset, we follow the previous literature [18], [19] in defining birth spacing as the number of months between the birth month for the child under observation and the birth month of the preceding birth. Covariates were selected based on a literature review and included: birth order, child age, child sex, multiple gestation, location of delivery, breastfeeding for the first six months of life, urban/rural residence, maternal education, mother's partner vital status, maternal partner education, household wealth quintile, and year of the DHS. Descriptive statistics for all covariates in both the maternal age and birth spacing dataset are presented in Table S2 in File S1. Household wealth quintiles were calculated by creating a wealth score based on ownership of materials and household characteristics based on principal component analysis as recommended by Filmer and Pritchett [25]. HAZ was computed from the crude child height and age data employing the Anthro Software package which utilizes the WHO Child Growth Standard [26]. Stunting was defined as a HAZ more than 2 standard deviations below the reference mean [27].

### Analysis

Log-poisson models were used to estimate relative risks for stunting employing the methodology of Zou [28]. Restricted cubic splines were first used to assess potential non-linear relationship of continuous maternal age and birth spacing with child stunting [29], [30]. To test for non-linearity, the likelihood ratio test was used to compare the model with only the linear term to the model with the linear and the cubic spline terms. We utilized the shape of the spline analysis with commonly used cut-offs to present a categorical analysis of maternal age (<18, 18–20, 20–26, 27–34, 35+ years) and birth spacing (<12, 12–23, 24–35, 36+ months). We also present a continuous analysis of birth spacing, since the spline analysis indicated a linear relationship.

*A priori* we decided to present stratified categorical analyses by sex, urban/rural residence, household wealth (poorest 50% vs. wealthiest 50%), and WHO World Bank region to assess heterogeneity in estimates. Potential modification of the maternal age association by birth order (firstborn versus birth order 2–5), birth spacing by birth order (birth order 2–5 versus 5+), and birth spacing by maternal age were also assessed. The Wald test for risk-ratio homogeneity was used to assess the statistical significance of the interaction. If significant effect modification was detected, stratified analyses were presented. As robustness check, multivariate linear regression models analyzing HAZ as a continuous outcome are also presented in the Tables S3 and S4 in File S1. All multivariate analyses included a fixed effect for each survey and the multivariate birth interval analysis also included categorical adjustment for maternal age. P-values for trend in categorical analyses were calculated by treating the median value of each maternal age or birth interval category as a continuous variable. P-values were two-sided with clustered robust standard errors to allow for local residual correlation as a result of the complex survey design utilized in DHS [31]. All regression analyses were conducted using STATA version 12 [32].

We then calculated the partial population-attributable risk percentage (PAR%) for teenage pregnancy and birth spacing <24 months by World Bank region for the DHS sample [33]. Partial PAR%s were calculated to estimate the percent of stunting cases that would not have occurred in the DHS sample if a hypothetical family planning intervention eliminated teen pregnancy and short birth intervals, but other risk factors for stunting did not change as a result of the intervention. We considered a hypothetical intervention which led all teenage pregnancies to occur at a maternal age of 20–26 years and all birth intervals <24 months to occur at 24–36 months. All region specific prevalences and effect sizes for other risk factors for stunting included in the multivariate model were assumed to remain constant in calculation of partial PAR%.

### Ethics Statement

De-identified secondary data was obtained through the Measure DHS website. The project involved no human subjects research.

## Results

### Sample Characteristics

The mean age at first birth across the 153 DHS was 20.4 years with 19% of all births occurring to teenage mothers (<20 years at birth). The DHS with the highest percentage of teenage pregnancies was Bangladesh with 34.8% in 2004, while the lowest was Rwanda in 2005 (6.8%). The median birth spacing interval for the sample was 33 months, with 21.7% of all births occurring less than 24 months from the preceding birth. The DHS with the highest percentage of births with an inter-pregnancy birth interval of less than 24 months was Jordan in 1990 (48.0%), while the lowest was Zimbabwe in 2010 (7.4%).

The covariate distribution among the total sample of children 6–59 months is summarized in Table S2 in File S1. Briefly, 49.4% of children were female, the mean child age was 30 months, 24.0% were firstborn children, 27.4% were of birth order 5 or higher, and the majority of children resided in rural areas (61.4%). As for mothers, 91.4% were married or living with a partner and 37.5% never attended any schooling. In terms of temporal coverage, 6.8% of children in the sample were born in the 1980s, 42.3% in the 1990s, and 50.9% in the 2000s.

### Maternal Age

**Figure 1:**
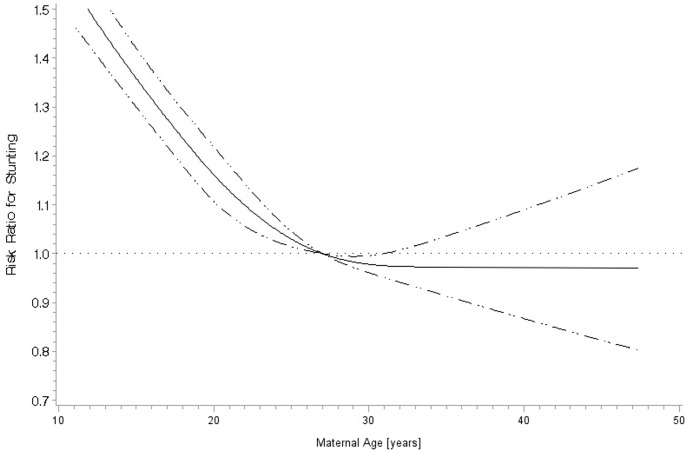
Non-linear adjusted^a^ relationship of maternal age with stunting for firstborn children^b^. ^a^Adjusted for same factors as Table 1 Caption. ^b^ 27 years is the reference group (p-value for non-linear relationship: <0.001).

There were 184,278 firstborn and 439,511 children of birth order 2–5 that contributed to the analysis of maternal age. The association of maternal age with stunting was significantly modified by birth order and as a result stratified analyses are presented (p-value for interaction: <0.001). The crude stunting prevalence for firstborn children was >50% for mothers reporting to be under 13 years of age and gradually declined to roughly 20% for mothers 27 years and older (Figure S5 in File S1). A multivariate restricted cubic spline analysis of continuous maternal age and stunting among firstborn children determined a significantly non-linear relationship (p-value for non-linear relationship: <0.001) which is presented in Figure 1. The estimated adjusted risk ratio for stunting declined gradually from a peak of 1.5 at age 13 years to the reference maternal age of 27 years (RR: 1.0). There was no indication of increased risk of stunting for maternal ages greater than 27 years, but statistical power was lacking due to low prevalence of first births among mothers in their thirties in LMICs. A similar relationship was found in a multivariate continuous analysis of maternal age and stunting among children of birth order 2–5, but the slope in risk of stunting was flatter for maternal ages less than the 27 year reference with maternal age less than 13 years carrying the greatest relative risk of 1.3 (not presented).

Table 1 shows the results of a multivariate categorical analysis of maternal age. The adjusted relative risk of stunting among firstborn children was 1.35 (95% CI: (1.30–1.40), 1.24 (95% CI: 1.19–1.29) and 1.15 (95% CI: 1.11–1.20) for maternal age groups <18, 18–19, and 20–26 years as compared to the reference group of mothers aged 27–34 years, respectively (p-value for trend: <0.001). Additional adjustment for birth spacing (a potential mediator) did not appear to reduce the strength of the association. Among children of birth order 2–5, there was also a significant association of maternal age with stunting, but the magnitude of the association was weaker (p-value for trend: <0.001) (Table 1). The adjusted relative risk of stunting among children birth order 2–5 was 1.20 (95% CI: (1.18–1.22), 1.14 (95% CI: 1.12–1.15) and 1.08 (95% CI: 1.06–1.09) for maternal age groups <18, 18–19, and 20–26 as compared to the 27–34 years reference, respectively. Secondary analysis of HAZ as a continuous outcome showed a similarly muted relationship of maternal age with stunting for children of birth order 2–5 as compared to firstborns (Table S3 in File S1).

**Table 1:** Association of maternal age with stunting for children aged 6–59 months by birth order, sex, household wealth, and World Bank region.

			Adjusted^a^ Relative Risk of Stunting by Maternal Age (95% CI)						
*Subgroup*	n	% Stunted	<18 years	18–19 years	20–26 years	27–34 years	35+ years	p-value for trend	p-value for interaction
Firstborn	184,278	35.0	1.38	1.27	1.16	1.0	0.99	<0.001	<0.001
			(1.33–1.43)	(1.22–1.31)	(1.12–1.20)	[Ref.]	(0.91–1.09)		
Birth order 2–5^b^	439,511	40.6	1.23	1.16	1.10	1.0	0.92	<0.001	
			(1.21–1.26)	(1.14–1.17)	(1.09–1.11)	[Ref.]	(0.90–0.93)		
***Among Firstborn Children***									
Males	93,171	36.8	1.35	1.24	1.14	1.0	0.96	<0.001	0.136
			(1.29–1.42)	(1.18–1.30)	(1.08–1.19)	[Ref.]	(0.85–1.09)		
Females	91,107	33.1	1.40	1.30	1.18	1.0	1.03	<0.001	
			(1.33–1.48)	(1.23–1.37)	(1.12–1.25)	[Ref.]	(0.90–1.18)		
Urban	78,907	24.1	1.58	1.38	1.22	1.0	0.91	<0.001	0.016
			(1.49–1.68)	(1.30–1.47)	(1.15–1.29)	[Ref.]	(0.78–1.07)		
Rural	105,371	43.1	1.26	1.18	1.09	1.0	1.05	<0.001	
			(1.21–1.32)	(1.13–1.23)	(1.05–1.14)	[Ref.]	(0.94–1.17)		
Poorest 50%	93,976	40.1	1.21	1.11	1.06	1.0	1.02	<0.001	<0.001
			(1.15–1.26)	(1.06–1.17)	(1.01–1.11)	[Ref.]	(0.91–1.14)		
Wealthiest 50%	90,302	29.6	1.56	1.42	1.25	1.0	0.94	<0.001	
			(1.48–1.65)	(1.34–1.50)	(1.18–1.32)	[Ref.]	(0.81–1.09)		
East Asia	3,782	46.3	1.23	1.17	1.11	1.0	0.98	0.001	<0.001
			(1.05–1.43)	(1.01–1.37)	(0.96–1.28)	[Ref.]	(0.73–1.31)		
Europe and	6,264	18.0	1.53	1.38	1.13	1.0	1.29	0.002	
Central Asia			(1.19–1.96)	(1.09–1.73)	(0.91–1.40)	[Ref.]	(0.84–1.98)		
Latin America	43,971	20.1	1.63	1.43	1.21	1.0	0.91	<0.001	
and Caribbean			(1.48–1.79)	(1.30–1.57)	(1.10–1.33)	[Ref.]	(0.74–1.14)		
Middle East and	18,465	23.7	1.09	1.11	1.07	1.0	0.87	0.025	
North Africa			(0.97–1.22)	(1.00–1.24)	(0.97–1.18)	[Ref.]	(0.66–1.15)		
South Asia	38,929	44.3	1.36	1.30	1.17	1.0	0.97	<0.001	
			(1.27–1.46)	(1.21–1.39)	(1.09–1.25)	[Ref.]	(0.79–1.18)		
Sub-Saharan	72,867	42.6	1.33	1.22	1.14	1.0	1.12	<0.001	
Africa			(1.26–1.41)	(1.15–1.29)	(1.08–1.21)	[Ref.]	(0.97–1.29)		

a Adjusted for child age in months, child sex, multiple birth, location of delivery, breastfeeding in first six months, rural residence, maternal education category, paternal education category, household wealth quintile, five-year period of birth, and survey fixed effects. Standard errors are clustered at the survey-cluster level to adjust for complex survey design used in the DHS data.

b Also adjusted for birth order.

In Table 1 stratified results of multivariate categorical models by child sex, urban/rural residence, household wealth, and World Bank region are also presented for firstborn children. There was no significant difference in the strength of association by sex (p-value for interaction: 0.136), but a significantly stronger association of maternal age with stunting was observed for children in urban areas as compared to rural (p-value for interaction: 0.016) and for households in the top 50% of household wealth as compared to bottom 50% (p-value for interaction: <0.001). The association of maternal age with stunting also significantly varied by WHO region (p-value <0.001). At the regional level, the strongest association between maternal age at first birth and stunting among firstborn children was found in the Latin America and Caribbean region, whereas the weakest was for the Middle East and North Africa region (MENA).

### Birth Spacing

**Figure 2:**
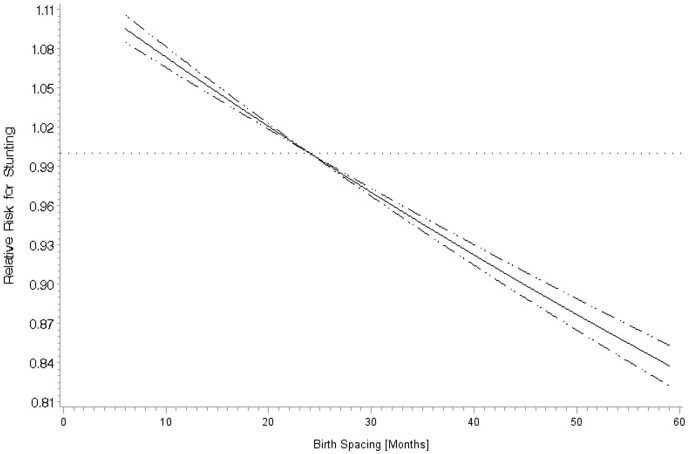
Linear adjusted^a^ relationship of birth spacing with stunting^b^ among children of birth order 2-5. ^a^ Adjusted for same factors as Table 1 Caption. ^b^ 24 months is the reference group (p-value for linear relationship: <0.001).

The sample size for birth spacing analyses was 584,226 children of birth order 2 or higher. Crude stunting prevalence was the highest for birth intervals less than 12 months (>40%) and gradually declined with increased birth interval length up to 60 months (5 years) (Figure S6 in File S1). Figure 2 shows the results of a multivariate restricted cubic spline analysis of birth spacing and stunting, which found a significantly linear relationship (p-value for linear relationship: <0.001). Similar to the crude data, the adjusted relative risk of stunting appeared to continuously decrease with increasing birth intervals and there was no indication of a plateau of the association.

In Table 2 results of multivariate categorical (<12, 12–23, 24–35, and ≥ 36months) and linear analyses of birth spacing are presented. The categorical analyses determined the relative risk of stunting for birth intervals <12 months and12–23 months were 1.09 (95% CI 1.06–1.12) and 1.06 (95% CI 1.05–1.06) as compared the reference of group 24–35 months, respectively. A birth interval of ≥36 months was associated with significantly decreased risk of stunting as compared to the 24–35 month reference group (RR: 0.91; 95% CI: 0.90–0.91) (p-value for trend: <0.001). In a multivariate linear analysis, each additional 6 months in the inter-pregnancy interval was associated with a 2.1% reduction in the relative risk of stunting (RR: 0.979; 95% CI: 0.977–0.979; p<0.001). Secondary analysis of HAZ score continuously found a similar relationship (Table S4 in File S1).

**Table 2:** Association of birth interval with stunting for children aged 6–59 months by birth order, sex, household wealth, and World Bank region.

			Adjusted^a^ Relative Risk of Stunting by Birth Interval (95% CI)					
*Sub Group*	n	% Stunted	<12 months	12–23 months	24–35 months	>36 months	p-value for trend	p-value for interaction
Birth order 2–5	439,511	40.6	1.11	1.06	1.0	0.89	<0.001	0.280
			(1.07–1.14)	(1.05–1.07)	[Ref.]	(0.89–0.90)		
Birth order 6+	144,715	47.8	1.05	1.05	1.0	0.90	<0.001	
			(1.00–1.10)	(1.03–1.06)	[Ref.]	(0.89–0.92)		
***Among birth order 2***–***5***								
Males	222,566	42.1	1.13	1.07	1.0	0.91	<0.001	0.100
			(1.08–1.18)	(1.05–1.08)	[Ref.]	(0.90–0.92)		
Females	216,945	39.0	1.08	1.06	1.0	0.88	<0.001	
			(1.03–1.12)	(1.04–1.07)	[Ref.]	(0.87–0.89)		
Urban	155,356	28.9	1.20	1.08	1.0	0.86	<0.001	0.506
			(1.13–1.27)	(1.06–1.11)	[Ref.]	(0.85–0.88)		
Rural	284,155	47.0	1.07	1.05	1.0	0.91	<0.001	
			(1.03–1.10)	(1.04–1.06)	[Ref.]	(0.90–0.92)		
Poorest 50%	247,845	45.1	1.06	1.05	1.0	0.91	<0.001	0.063
			(1.03–1.11)	(1.04–1.06)	[Ref.]	(0.90–0.92)		
Top 50%	191,666	34.8	1.17	1.08	1.0	0.88	<0.001	
			(1.11–1.23)	(1.06–1.09)	[Ref.]	(0.87–0.89)		
East Asia	9,796	52.2	1.15	1.03	1.0	0.90	<0.001	<0.001
			(0.95–1.39)	(0.98–1.08)	[Ref.]	(0.86–0.94)		
Europe and	9,936	43.7	1.35	1.07	1.0	0.86	<0.001	
Central Asia			(1.07–1.71)	(0.99–1.17)	[Ref.]	(0.79–0.94)		
Latin America	88,086	28.6	1.21	1.06	1.0	0.77	<0.001	
and Caribbean			(1.12–1.31)	(1.03–1.08)	[Ref.]	(0.75–0.79)		
Middle East and	45,101	26.5	1.22	1.12	1.0	0.91	<0.001	
North Africa			(1.10–1.36)	(1.08–1.16)	[Ref.]	(0.87–0.94)		
South Asia	80,059	52.5	1.09	1.05	1.0	0.91	<0.001	
			(1.03–1.14)	(1.03–1.06)	[Ref.]	(0.90–0.93)		
Sub-Saharan	206,533	44.4	1.04	1.06	1.0	0.92	<0.001	
Africa			(0.99–1.09)	(1.04–1.07)	[Ref.]	(0.91–0.93)		

a Adjusted for maternal age, birth order, child age in months, child sex, multiple birth, location of delivery, breastfeeding in first six months, rural residence, maternal education category, paternal education category, household wealth quintiles, five-year period of birth, and survey fixed effects. Standard errors are clustered at the survey-cluster level to adjust for complex survey design used in the DHS data.

Stratified results of multivariate categorical models for birth spacing by birth order, sex, urban/rural residence, household wealth, and World Bank region are also presented in Table 2. There was no significant difference in the strength of the association of birth spacing with stunting by birth order, child sex, or household wealth (all p-value for interaction >0.05). Nevertheless, there was significant heterogeneity in the association by WHO region (p-value for interaction <0.001).

### Estimated Population-Level Impact

Due to substantial variation in both the estimated relative risk and the prevalence of teenage pregnancy, the estimated population impact of eliminating teenage pregnancies in the DHS sample varied widely by WHO region. As Table 3 shows, in the South Asian DHS sample an estimated 6.9% (6.2–7.6%) of stunting cases could have been averted by eliminating teenage pregnancies, while the same is true for only 0.8% of stunting cases (0–1.6%) in the MENA region. The percentage of stunting cases attributed to birth intervals <24 months was relatively similar across region. The highest PAR% estimate for short birth intervals was observed for the MENA region (3.0%; 95% CI: 1.7–4.2%), while the lowest was determined for Latin America and the Caribbean (1.2%; 95% CI: 0.2–2.1%). We also estimate that by eliminating both teenage pregnancy and birth intervals <24 months, 8.6% (6.9–10.3%) of stunting cases could have been averted in the South Asian DHS sample, while only 3.6% (95% CI: 1.5–5.7) would have been prevented in the MENA region sample.

**Table 3:** Estimated percent reduction in stunted children by eliminating teenage pregnancy and birth intervals <24 months* by World Bank region within DHS sample.

	% of births occurring to teenage mothers	% of births occurring <24 months birth spacing	Partial PAR% Teenage pregnancy	Partial PAR% <24 months birth spacing	Partial PAR% Teenage Pregnancy and <24 months birth spacing
East Asia and Pacific	10.6	18.4	2.3 (1.0–3.5)	2.2 (−0.2–4.6)	4.3 (0.6–7.9)
Europe and Central Asia	16.4	18.5	5.3 (3.9–6.7)	1.8 (−1.7–5.3)	6.6 (1.4–11.8)
Latin America and the Caribbean	21.5	17.8	5.2 (4.7–5.8)	1.2 (0.2–2.1)	5.6 (4.0–7.1)
Middle East and North Africa	11.9	21.9	0.8 (0–1.6)	3.0 (1.7–4.2)	3.6 (1.5–5.7)
South Asia	23.8	16.3	6.9 (6.2–7.6)	2.3 (1.5–3.2)	8.6 (6.9–10.3)
Sub-Saharan Africa	18.3	14.6	3.8 (3.4–4.3)	2.0 (1.6–2.4)	5.4 (4.5–6.2)

PAR% =  Population attributable risk % or the % of stunting cases that can be attributed to the risk factor(s) of interest.

* Assuming all teenage pregnancies would occur at a maternal age of 20–26 years and all birth intervals <24 months would occur at 24–36 month intervals. Regional specific also used in calculation of partial PAR%.

## Discussion

The individual and population level analyses of birth timing presented in this work have yielded several key findings. Foremost, young maternal age at first birth is a substantial risk factor for child stunting, while the association of short birth intervals with restricted linear growth appears to be weak. In terms of the shape of these relationships, the risk of stunting was highest for maternal ages under 18 years with declines in risk up to 27 years. As for birth spacing, the highest risk of stunting was observed for birth intervals of less than 12 months with gradual linear decreases in risk for longer birth intervals.

In the DHS sample stunting rates are substantially lower for firstborn children as compared to children of higher birth order, while the reverse is true for infant mortality [4]. In the British context, firstborn children were shown to be smaller at birth but then exhibited rapid catch-up growth and reached greater heights as compared to higher birth order children by 12 months of age [34]. A similar growth catch-up mechanism may partially explain the low prevalence of stunting for firstborn children in LMICs. Most studies from developing countries have found the association of maternal age with child mortality to be weak [4], [35], whereas the relative risk of child mortality appears to sharply increase for birth intervals of less than 18 months [18], [19]. Our results suggest the opposite is true for linear growth, that there is a relatively small increase in the risk of stunting associated with short birth intervals, while the risk of stunting is substantial for children born to teenage mothers. As a result, the mechanisms underlying the observed relationships of birth timing with mortality and physical growth are likely to be different. It is possible the biological factors which lead to a generally strong association of short birth intervals with early infant mortality and reduced birth size are not as significant contributors to childhood stunting due to the potential for growth catch-up [34], [36], [37]. This is in contrast to the social, economic, and behavioral consequences of young maternal age which may persist as key drivers of physical growth throughout childhood.

Our results also suggest remarkable heterogeneity in the strength of the maternal age and stunting association across socioeconomic groups and by urban/rural residence. Even though we hypothesized *a priori* there would be significant heterogeneity in the association of birth timing with stunting, the finding that young maternal age at first birth is relatively more harmful in urban and in wealthier households as compared to rural and poorer households was not anticipated. This finding is partially driven by the use of relative risk measures in our primary analysis, as the significantly lower prevalence of stunting in urban and wealthy households may result in the same absolute increase in the probability of stunting yielding a larger observed relative risk. Nevertheless, the heterogeneous relationship remained when analyzing HAZ continuously (Tables S3 and S4 in File S1). One potential mechanism for the observed effect modification is that income differentials between older and younger mothers are more pronounced in urban and wealthier strata and the relatively simple asset score used by DHS does not completely capture these differences. An alternative explanation is that in rural areas and among poorer households stunting may be primarily the result of inadequate food availability and variety [38], micronutrient deficiency [39], or poor sanitation [40] while having a young mother may not be as important of a factor for children facing significant nutritional and environmental adversity. The relatively higher impact in urban strata may also reflect the relatively high risk faced by young mothers in urban slum neighborhoods, which we cannot directly identify in the DHS data, and which may appear relatively wealthy in asset-based indices. Independent of the mechanisms driving this heterogeneity, it seems likely that the relative importance of young maternal age as a population-level risk factor for stunting will increase over the coming years as LMICs become increasingly urbanized and also develop economically [41].

The associations of maternal age and birth spacing with child stunting also varied substantially across WHO region, which may reflect differences in wealth and urbanization along with other regional factors like social support and family structure, prevalence of childhood infections, and food security.

Overall, our results suggest that the combined burden of teenage motherhood and short birth intervals is largest for the South Asian region, where we estimate that close to 9% of stunting cases could be averted with improved birth spacing, followed by Europe and Central Asia (6.6%), Latin America and the Caribbean (5.6%), Sub-Saharan Africa (5.4%), East Asia and Pacific (4.3%) and MENA (3.6%). The larger impact in the first three regions is primarily the result of their high prevalence of teenage motherhood, which is relatively rare in the East Asia and MENA regions. In terms of birth spacing, largest improvements seem possible for the MENA region, where more than one in five children are born within less than 24 months of the preceding birth.

A primary concern in the interpretation of DHS analyses is the cross-sectional nature of the data. While reverse causality concerns are often salient in cross-sectional studies, the potential for reverse causation should be minimal in this analysis due to the known temporal ordering of events. Nevertheless, residual or unmeasured confounding is possible. Residual confounding by socioeconomic status may be of particular importance because household asset ownership may not completely capture relative economic standing, especially for households in urban slum areas. More generally, birth timing decisions are the result of a complex set of individual, social, and other contextual factors, whose omission could potentially bias the results presented, so that the estimated associations may not necessarily reflect the true causal effect of interest.

The results presented in this study suggest that young maternal age and short birth intervals are risk factors for restricted linear growth, which implies that lowering adolescent fertility and increasing birth intervals has the potential to substantially reduce the number of stunted children, particularly for the South Asian region. Even though birth timing is the result of a complex combination of biological, social, and behavioral factors [3], [5], [18], [20], [22], [23], [35], [42], [43], large reductions in adolescent fertility [44] and short birth intervals [21] through increased availability and use of contraceptives seems possible. More than 900 million women are estimated to still face unmet needs for contraception globally [45], and the potential improvements in child physical growth shown in this paper provide further evidence in support of expansion of family planning services.

## Supporting Information

File S1 Contains the following files: Table S1: Survey List. Table S2. Covariate distribution for Maternal Age (n = 623,789) and Birth Interval (n = 584,226) Datasets. Table S3. Association of Maternal Age with HAZ for Children Aged 6-36. Table S4. Association of Birth Spacing with HAZ for Children Aged 6-36. Figure S4: Geographical Coverage of 61 Sample Countries. Figure S5. Crude stunting prevalence for first born children by maternal age. Figure S6. Crude stunting prevalence for first born children by birth interval.
(DOCX)

## References

[1] WHO (2011) WHO guidelines on preventing early pregnancy and poor reproductive health outcomes among adolescents in developing countries. Geneva: WHO. 26180870

[2] Mayor S (2004) Pregnancy and childbirth are leading causes of death in teenage girls in developing countries. BMJ 328: 1152. 10.1136/bmj.328.7449.1152-a PMC411126 15142897

[3] Alam N (2000) Teenage motherhood and infant mortality in Bangladesh: maternal age-dependent effect of parity one. J Biosoc Sci 32: 229–236. 10765612 10.1017/s0021932000002297

[4] Finlay JE, Ozaltin E, Canning D (2011) The association of maternal age with infant mortality, child anthropometric failure, diarrhoea and anaemia for first births: evidence from 55 low- and middle-income countries. BMJ Open 1: e000226. 10.1136/bmjopen-2011-000226 PMC3191600 22021886

[5] Raj A, Saggurti N, Winter M, Labonte A, Decker MR, et al (2010) The effect of maternal child marriage on morbidity and mortality of children under 5 in India: cross sectional study of a nationally representative sample. BMJ 340: b4258. 20093277 10.1136/bmj.b4258 PMC2809839

[6] Gupta N, Kiran U, Bhal K (2008) Teenage pregnancies: obstetric characteristics and outcome. Eur J Obstet Gynecol Reprod Biol 137: 165–171. 17900787 10.1016/j.ejogrb.2007.06.013

[7] Guimaraes AM, Bettiol H, Souza LD, Gurgel RQ, Almeida ML, et al (2013) Is adolescent pregnancy a risk factor for low birth weight? Rev Saude Publica 47: 11–19. 23703125 10.1590/s0034-89102013000100003

[8] Zabin LS, Kiragu K (1998) The health consequences of adolescent sexual and fertility behavior in sub-Saharan Africa. Stud Fam Plann 29: 210–232. 9664633

[9] Senderowitz J, Paxman JM (1985) Adolescent fertility: worldwide concerns. Popul Bull 40: 1–51. 12340104

[10] Chen XK, Wen SW, Fleming N, Demissie K, Rhoads GG, et al (2007) Teenage pregnancy and adverse birth outcomes: a large population based retrospective cohort study. Int J Epidemiol 36: 368–373. 17213208 10.1093/ije/dyl284

[11] Shaw M, Lawlor DA, Najman JM (2006) Teenage children of teenage mothers: psychological, behavioural and health outcomes from an Australian prospective longitudinal study. Soc Sci Med 62: 2526–2539. 16332404 10.1016/j.socscimed.2005.10.007

[12] Coyne CA, Langstrom N, Lichtenstein P, D'Onofrio BM (2013) The association between teenage motherhood and poor offspring outcomes: a national cohort study across 30 years. Twin Res Hum Genet 16: 679–689. 23632141 10.1017/thg.2013.23 PMC3657321

[13] Stevens GA, Finucane MM, Paciorek CJ, Flaxman SR, White RA, et al (2012) Trends in mild, moderate, and severe stunting and underweight, and progress towards MDG 1 in 141 developing countries: a systematic analysis of population representative data. Lancet 380: 824–834. 22770478 10.1016/S0140-6736(12)60647-3 PMC3443900

[14] Grantham-McGregor S, Cheung YB, Cueto S, Glewwe P, Richter L, et al (2007) Developmental potential in the first 5 years for children in developing countries. Lancet 369: 60–70. 17208643 10.1016/S0140-6736(07)60032-4 PMC2270351

[15] Kar BR, Rao SL, Chandramouli BA (2008) Cognitive development in children with chronic protein energy malnutrition. Behav Brain Funct 4: 31. 18652660 10.1186/1744-9081-4-31 PMC2519065

[16] Heckman J, Stixrud J, Urzua S (2006) The effects of cognitive and noncognitive abilities on labor market outcomes and social behavior. Journal of Labor Economics 24: 411–482.

[17] Heckman JJ (2007) The Economics, Technology, and Neuroscience of Human Capability Formation. Proceedings of the National Academy of Sciences 104: 13250–13255. 10.1073/pnas.0701362104 PMC1948899 17686985

[18] Rutstein SO (2005) Effects of preceding birth intervals on neonatal, infant and under-five years mortality and nutritional status in developing countries: evidence from the demographic and health surveys. Int J Gynaecol Obstet 89 Suppl 1S7–24. 15820369 10.1016/j.ijgo.2004.11.012

[19] Rutstein SO (2008) Further evidence of the effects of preceding birth intervals on neonatal, infant, and under-five-years mortality and nutritional status in developing countries: Evidence from the demographic health surveys. DHS Working Paper 41. 10.1016/j.ijgo.2004.11.012 15820369

[20] Adhikari R (2003) Early marriage and childbearing: risks and consequences. In: Bott S, Jejeebhoy S, Shah I, Puriet C, editors. Towards adulthood: exploring the sexual and reproductive health of adolescents in South Asia: World Health Organization:. pp. 62–66.

[21] Alvergne A, Lawson DW, Clarke PM, Gurmu E, Mace R (2013) Fertility, parental investment, and the early adoption of modern contraception in rural Ethiopia. Am J Hum Biol 25: 107–115. 23180659 10.1002/ajhb.22348

[22] Reynolds HW, Wong EL, Tucker H (2006) Adolescents' use of maternal and child health services in developing countries. Int Fam Plan Perspect 32: 6–16. 16723297 10.1363/3200606

[23] Conde-Agudelo A, Rosas-Bermudez A, Castano F, Norton MH (2012) Effects of birth spacing on maternal, perinatal, infant, and child health: a systematic review of causal mechanisms. Stud Fam Plann 43: 93–114. 23175949 10.1111/j.1728-4465.2012.00308.x

[24] World Bank (2012) World Development Indicators Online database.

[25] Filmer D, Pritchett LH (2001) Estimating wealth effects without expenditure data - or tears: An application to educational enrollments in states of India. Demography 38: 115–132. 11227840 10.1353/dem.2001.0003

[26] WHO (2006) Anthro Software for assessing growth and development of the world's children. Geneva: WHO.

[27] WHO Multicentre Growth Reference Study Group (2006) WHO Child Growth Standards: Length/height-for-age, weight-for-age, weight-for-length, weight-for-height and body mass index-for-age: Methods and development. Geneva: World Health Organization.

[28] Zou G (2004) A Modified Poisson Regression Approach to Prospective Studies with Binary Data. American Journal of Epidemiology 159: 702–706. 15033648 10.1093/aje/kwh090

[29] Durrleman S, Simon R (1989) Flexible regression models with cubic splines. Stat Med 8: 551–561. 2657958 10.1002/sim.4780080504

[30] Govindarajulu US, Spiegelman D, Thurston SW, Ganguli B, Eisen EA (2007) Comparing smoothing techniques in Cox models for exposure-response relationships. Stat Med 26: 3735–3752. 17538974 10.1002/sim.2848

[31] ICF International (2012) Demographic and Health Survey - Sampling and Household Listing Manual In: MEASURE DHS, editor. Calverton, Maryland USA.

[32] StataCorp (2011) Stata Statistical Software: Release 12. College Station, TX: StataCorp LP.

[33] Spiegelman D, Hertzmark E, Wand HC (2007) Point and interval estimates of partial population attributable risks in cohort studies: examples and software. Cancer Causes Control 18: 571–579. 17387622 10.1007/s10552-006-0090-y

[34] Ong KK, Preece MA, Emmett PM, Ahmed ML, Dunger DB (2002) Size at birth and early childhood growth in relation to maternal smoking, parity and infant breast-feeding: longitudinal birth cohort study and analysis. Pediatr Res 52: 863–867. 12438662 10.1203/00006450-200212000-00009

[35] Scally G (2002) Too much too young? Teenage pregnancy is a public health, not a clinical, problem. Int J Epidemiol 31: 554–555. 12055152 10.1093/ije/31.3.554

[36] Prentice AM, Ward KA, Goldberg GR, Jarjou LM, Moore SE, et al (2013) Critical windows for nutritional interventions against stunting. Am J Clin Nutr 97: 911–918. 23553163 10.3945/ajcn.112.052332 PMC3628381

[37] Wells JCK, Hallal PC, Reichert FF, Dumith SC, Menezes AM, et al (2011) Associations of Birth Order With Early Growth and Adolescent Height, Body Composition, and Blood Pressure: Prospective Birth Cohort From Brazil. American Journal of Epidemiology 174: 1028–1035. 21940799 10.1093/aje/kwr232 PMC3658103

[38] UNICEF (1990) Strategy for improved nutrition of children and women in developing countries. New York, NY: UNICEF. 10.1007/BF02810402 1937618

[39] Caulfield LE, Richard SA, Rivera JA, Musgrove P, Black RE (2006) Stunting, Wasting, and Micronutrient Deficiency Disorders. In: Jamison D, Breman J, Measham A, editors. Disease Control Priorities in Developing Countries. Washington (DC): World Bank. 21250337

[40] Fink G, Günther I, Hill K (2011) The effect of water and sanitation on child health: evidence from the demographic and health surveys 1986–2007. Int J Epidemiol 40: 1196–1204. 21724576 10.1093/ije/dyr102

[41] Bloom DE, Canning D, Fink G, Khanna T, Salyer P (2010) Urban Settlement: Data, Measures, and Trends. In: Beall J, Huha-Khasnobis B, Kanbur R, editors. Urbanization and Development: Multidisciplinary Perspectives: Oxford University Press.

[42] King JC (2003) The risk of maternal nutritional depletion and poor outcomes increases in early or closely spaced pregnancies. J Nutr 133: 1732S–1736S. 12730491 10.1093/jn/133.5.1732S

[43] Stewart CP, Katz J, Khatry SK, LeClerq SC, Shrestha SR, et al (2007) Preterm delivery but not intrauterine growth retardation is associated with young maternal age among primiparae in rural Nepal. Matern Child Nutr 3: 174–185. 17539886 10.1111/j.1740-8709.2007.00097.x PMC2367231

[44] Yen S, Martin S (2013) Contraception for adolescents. Pediatr Ann 42: 21–25. 23379400 10.3928/00904481-20130128-08

[45] Alkema L, Kantorova V, Menozzi C, Biddlecom A (2013) National, regional, and global rates and trends in contraceptive prevalence and unmet need for family planning between 1990 and 2015: a systematic and comprehensive analysis. Lancet 381: 1642–1652. 23489750 10.1016/S0140-6736(12)62204-1

