# Supplementary material for: Scaling-Up Access to Family Planning May Improve Linear Growth and Child Development in Low and Middle Income Countries
Source: PLoS One. 2014 Jul 14;9(7):e102391. doi: 10.1371/journal.pone.0102391 (PMC4096753; doi:10.1371/journal.pone.0102391)
Supplement: File S1 — Contains the following files: Table S1: Survey List. Table S2. Covariate distribution for Maternal Age (n = 623,789) and Birth Interval (n = 584,226) Datasets. Table S3. Association of Maternal Age with HAZ for Children Aged 6-36. Table S4. Association of Birth Spacing with HAZ for Children Aged 6-36. Figure S4: Geographical Coverage of 61 Sample Countries. Figure S5. Crude stunting prevalence for first born children by maternal age. Figure S6. Crude stunting prevalence for first born children by birth interval. (DOCX) [file pone.0102391.s001.docx]

**Supporting Information Table S1: Survey List**

**Supporting Information Table S2. Covariate distribution for Maternal Age (n=623,789) and Birth Interval (n=584,226) Datasets**

|  | Maternal Age Dataset | | Birth Interval Dataset | |
| --- | --- | --- | --- | --- |
| Variable | n | % or SD | n |  |
| First born child, N (%) | 184,278 | (29.54) |  |  |
| Parity 2-4, N (%) | 373,631 | (59.90) | 373,631 | (63.95) |
| Parity 5 or higher, N (%) | 65,880 | (10.56) | 210,595 | (36.05) |
| Age of child in months, Mean (SD) | 30.4 | (15.46) | 30.7 | (15.46) |
| Child is female, N (%) | 308,052 | (49.38) | 288,742 | (49.42) |
| Child is multiple birth, N (%) | 11,388 | (1.83) | 15,487 | (2.65) |
| Child delivered at home, N (%) | 286,765 | (45.97) | 314,790 | (53.88) |
| Breastfeeding first six months, N (%) | 545,629 | (87.47) | 522,212 | (89.39) |
| Rural residence, N(%) | 389,526 | (62.45) | 395,270 | (67.66) |
| Mother no education, N (%) | 204,092 | (32.72) | 244,435 | (41.84) |
| Mother primary education, N (%) | 211,907 | (33.97) | 203,701 | (34.87) |
| Mother secondary education, N (%) | 171,874 | (27.55) | 115,847 | (19.83) |
| Mother tertiary education, N (%) | 35,916 | (5.76) | 20,243 | (3.46) |
| Mother married or living with partner, N(%) | 565,174 | (90.60) | 545,441 | (93.36) |
| Partner no education, N (%) | 145,237 | (23.28) | 176,300 | (30.18) |
| Partner primary education, N (%) | 191,231 | (30.66) | 197,491 | (33.80) |
| Partner secondary education, N (%) | 195,343 | (31.32) | 150,481 | (25.76) |
| Partner tertiary education, N (%) | 53,885 | (8.64) | 36,590 | (6.26) |
| Household has electricity, N (%) | 277,414 | (44.47) | 221,219 | (37.87) |
| Household has TV, N (%) | 240,222 | (38.51) | 189,536 | (32.44) |
| Household has fridge, N (%) | 140,392 | (22.51) | 106,136 | (18.17) |
| Household has motor bike, N (%) | 171,427 | (27.48) | 166,197 | (28.45) |
| Household has car, N (%) | 62,516 | (10.02) | 53,799 | (9.21) |
| Child born in the 1980s, N (%) | 38,458 | (6.17) | 42,353 | (7.25) |
| Child born1990-1994, N (%) | 107,172 | (17.18) | 105,030 | (17.98) |
| Child born 1995-1999, N (%) | 152,111 | (24.39) | 143,770 | (24.61) |
| Child born 2000-2004, N (%) | 185,643 | (29.76) | 167,267 | (28.63) |
| Child born 2005-2009, N (%) | 128,745 | (20.64) | 115,365 | (19.75) |
| Child born 2010 or later, N (%) | 11,660 | (1.87) | 10,441 | (1.79) |

**Supporting Information Table S3. Association of Maternal Age with HAZ for Children Aged 6-36**

|  |  |  | **Adjusted^a^ HAZ Differentials by Maternal Age (95% CI)** | | | | |  |  |
| --- | --- | --- | --- | --- | --- | --- | --- | --- | --- |
| ***Subgroup*** | n | Mean HAZ | <18 years | 18-19 years | 20-26 years | 27-34 years | 35+ years | p-value for trend | p-value for interaction |
| First Born | 184,278 | -1.43 | -0.41 | -0.27 | -0.15 | 1.0 | 0.07 | <0.001 | <0.001 |
|  |  |  | (-0.44 - -0.38) | (-0.31 - -0.24) | (-0.18 - -0.13) | [Ref.] | (-0.00 - 0.15) |  |  |
| Birth order 2-5^b^ | 439,511 | -1.63 | -0.36 | -0.24 | -0.13 | 1.0 | 0.12 | <0.001 |  |
|  |  |  | (-0.39 - -0.33) | (-0.26 - -0.22) | (-0.14 - -0.12) | [Ref.] | (0.10 - 0.14) |  |  |
| ***Among First Born Children*** | | |  |  |  |  |  |  |  |
| Males | 93,171 | -1.49 | -0.44 | -0.29 | -0.17 | 1.0 | 0.05 | <0.001 | 0.002 |
|  |  |  | (-0.49 - -0.40) | (-0.34 - -0.24) | (-0.21 - -0.13) | [Ref.] | (-0.05 - 0.16) |  |  |
| Females | 91,107 | -1.37 | -0.37 | -0.26 | -0.14 | 1.0 | 0.10 | <0.001 |  |
|  |  |  | (-0.41 - -0.33) | (-0.30 - -0.21) | (-0.18 - -0.10) | [Ref.] | (-0.01 - 0.21) |  |  |
| Urban | 78,907 | -1.05 | -0.46 | -0.31 | -0.18 | 1.0 | 0.09 | <0.001 | 0.087 |
|  |  |  | (-0.50 - -0.42) | (-0.35 - -0.27) | (-0.21 - -0.14) | [Ref.] | (-0.00 - 0.18) |  |  |
| Rural | 105,371 | -1.72 | -0.35 | -0.23 | -0.11 | 1.0 | 0.05 | <0.001 |  |
|  |  |  | (-0.40 - -0.30) | (-0.28 - -0.17) | (-0.16 - -0.06) | [Ref.] | (-0.08 - 0.18) |  |  |
| Poorest 50% | 93,976 | -1.60 | -0.31 | -0.17 | -0.09 | 1.0 | 0.06 | <0.001 | <0.001 |
|  |  |  | (-0.36 - -0.26) | (-0.22 - -0.13) | (-0.14 - -0.04) | [Ref.] | (-0.05 - 0.17) |  |  |
| Wealthiest 50% | 90,302 | -1.26 | -0.46 | -0.34 | -0.19 | 1.0 | 0.09 | <0.001 |  |
|  |  |  | (-0.50 - -0.42) | (-0.38 - -0.29) | (-0.22 - -0.15) | [Ref.] | (-0.01 - 0.19) |  |  |
| East Asia | 3,782 | -1.84 | -0.42 | -0.23 | -0.14 | 1.0 | 0.15 | <0.001 | <0.001 |
|  |  |  | (-0.64 - -0.21) | (-0.44 - -0.03) | (-0.32 - 0.04) | [Ref.] | (-0.21 - 0.50) |  |  |
| Europe and | 6,264 | -0.78 | -0.39 | -0.26 | -0.17 | 1.0 | -0.23 | <0.001 |  |
| Central Asia |  |  | (-0.55 - -0.23) | (-0.40 - -0.12) | (-0.30 - -0.05) | [Ref.] | (-0.58 - 0.12) |  |  |
| Latin America | 43,971 | -0.97 | -0.43 | -0.29 | -0.15 | 1.0 | 0.13 | <0.001 |  |
| and Caribbean |  |  | (-0.48 - -0.38) | (-0.34 - -0.25) | (-0.19 - -0.10) | [Ref.] | (0.03 - 0.22) |  |  |
| Middle East and | 18,465 | -0.97 | -0.21 | -0.20 | -0.13 | 1.0 | 0.22 | <0.001 |  |
| North Africa |  |  | (-0.31 - -0.10) | (-0.30 - -0.10) | (-0.22 - -0.05) | [Ref.] | (-0.00 - 0.45) |  |  |
| South Asia | 38,929 | -1.76 | -0.41 | -0.30 | -0.14 | 1.0 | 0.12 | <0.001 |  |
|  |  |  | (-0.48 - -0.33) | (-0.37 - -0.22) | (-0.21 - -0.07) | [Ref.] | (-0.09 - 0.33) |  |  |
| Sub-Saharan | 72,867 | -1.69 | -0.42 | -0.27 | -0.16 | 1.0 | -0.11 | <0.001 |  |
| Africa |  |  | (-0.48 - -0.35) | (-0.33 - -0.20) | (-0.23 - -0.10) | [Ref.] | (-0.29 - 0.08) |  |  |
| ^a^ Adjusted for child age in months, child sex, multiple birth, location of delivery, breastfeeding in first six months, rural residence, maternal education category, paternal education category, indictors for household wealth quintiles 2-5, five-year period of birth, and survey fixed effects. Standard errors are clustered at the survey-cluster level to adjust for complex survey design used in the DHS data.  ^b^ Also adjusted for birth order | | | | | | | | | |

**Supporting Information Table S4. Association of Birth Spacing with HAZ for Children Aged 6-36**

|  |  |  | **Adjusted^a^ HAZ Differentials by Birth Interval (95% CI)** | | | |  |  |
| --- | --- | --- | --- | --- | --- | --- | --- | --- |
| ***Sub Group*** | n | Mean HAZ | <12 months | 12-23 months | 24-35 months | 36+ months | p-value for trend | p-value for interaction |
| Birth order 2-5 | 439,511 | -1.63 | -0.17 | -0.10 | 1.0 | 0.17 | <0.001 | 0.280 |
|  |  |  | (-0.22 - -0.12) | (-0.11 - -0.09) | [Ref.] | (0.16 - 0.18) |  |  |
| Birth order 6+ | 144,715 | -1.87 | -0.08 | -0.08 | 1.0 | 0.18 | <0.001 |  |
|  |  |  | (-0.17 - 0.00) | (-0.11 - -0.06) | [Ref.] | (0.17 - 0.20) |  |  |
| ***Among Birth Order 2-5 Children*** | | |  |  |  |  |  |  |
| Males | 222,566 | -1.68 | -0.20 | -0.11 | 1.0 | 0.16 | <0.001 | 0.868 |
|  |  |  | (-0.27 - -0.13) | (-0.13 - -0.09) | [Ref.] | (0.15 - 0.18) |  |  |
| Females | 216,945 | -1.57 | -0.13 | -0.09 | 1.0 | 0.17 | <0.001 |  |
|  |  |  | (-0.20 - -0.06) | (-0.11 - -0.07) | [Ref.] | (0.16 - 0.19) |  |  |
| Urban | 155,356 | -1.22 | -0.23 | -0.10 | 1.0 | 0.17 | <0.001 | 0.378 |
|  |  |  | (-0.31 - -0.15) | (-0.12 - -0.08) | [Ref.] | (0.15 - 0.19) |  |  |
| Rural | 284,155 | -1.84 | -0.13 | -0.10 | 1.0 | 0.17 | <0.001 |  |
|  |  |  | (-0.19 - -0.07) | (-0.11 - -0.08) | [Ref.] | (0.15 - 0.18) |  |  |
| Poorest 50% | 247,845 | -1.77 | -0.13 | -0.09 | 1.0 | 0.17 | <0.001 | 0.332 |
|  |  |  | (-0.19 - -0.06) | (-0.11 - -0.08) | [Ref.] | (0.15 - 0.18) |  |  |
| Top 50% | 191,666 | -1.43 | -0.22 | -0.10 | 1.0 | 0.16 | <0.001 |  |
|  |  |  | (-0.30 - -0.15) | (-0.12 - -0.08) | [Ref.] | (0.14 - 0.18) |  |  |
| East Asia and | 9,796 | -1.98 | -0.09 | -0.03 | 1.0 | 0.24 | <0.001 | <0.001 |
| Pacific |  |  | (-0.52 - 0.34) | (-0.12 - 0.06) | [Ref.] | (0.16 - 0.32) |  |  |
| Europe and | 9,936 | -1.01 | -0.45 | -0.12 | 1.0 | 0.14 | <0.001 |  |
| Central Asia |  |  | (-0.73 - -0.17) | (-0.21 - -0.04) | [Ref.] | (0.06 - 0.21) |  |  |
| Latin America | 88,086 | -1.28 | -0.19 | -0.08 | 1.0 | 0.23 | <0.001 |  |
| and Caribbean |  |  | (-0.28 - -0.09) | (-0.10 - -0.05) | [Ref.] | (0.21 - 0.25) |  |  |
| Middle East and | 45,101 | -1.10 | -0.24 | -0.12 | 1.0 | 0.15 | <0.001 |  |
| North Africa |  |  | (-0.36 - -0.12) | (-0.16 - -0.08) | [Ref.] | (0.11 - 0.19) |  |  |
| South Asia | 80,059 | -2.04 | -0.20 | -0.10 | 1.0 | 0.16 | <0.001 |  |
|  |  |  | (-0.31 - -0.10) | (-0.13 - -0.07) | [Ref.] | (0.14 - 0.19) |  |  |
| Sub-Saharan | 206,533 | -1.74 | -0.09 | -0.10 | 1.0 | 0.14 | <0.001 |  |
| Africa |  |  | (-0.17 - -0.01) | (-0.12 - -0.08) | [Ref.] | (0.12 - 0.16) |  |  |

^a^ Adjusted for child age in months, child sex, multiple birth, location of delivery, breastfeeding in first six months, rural residence, maternal education category, paternal education category, indicators for household wealth quintiles 2-5, five-year period of birth, and survey fixed effects. Standard errors are clustered at the survey-cluster level to adjust for complex survey design used in the DHS data.

^b^ Also adjusted for birth order

**Supporting Information Figure S4: Geographical Coverage of 61 Sample Countries**


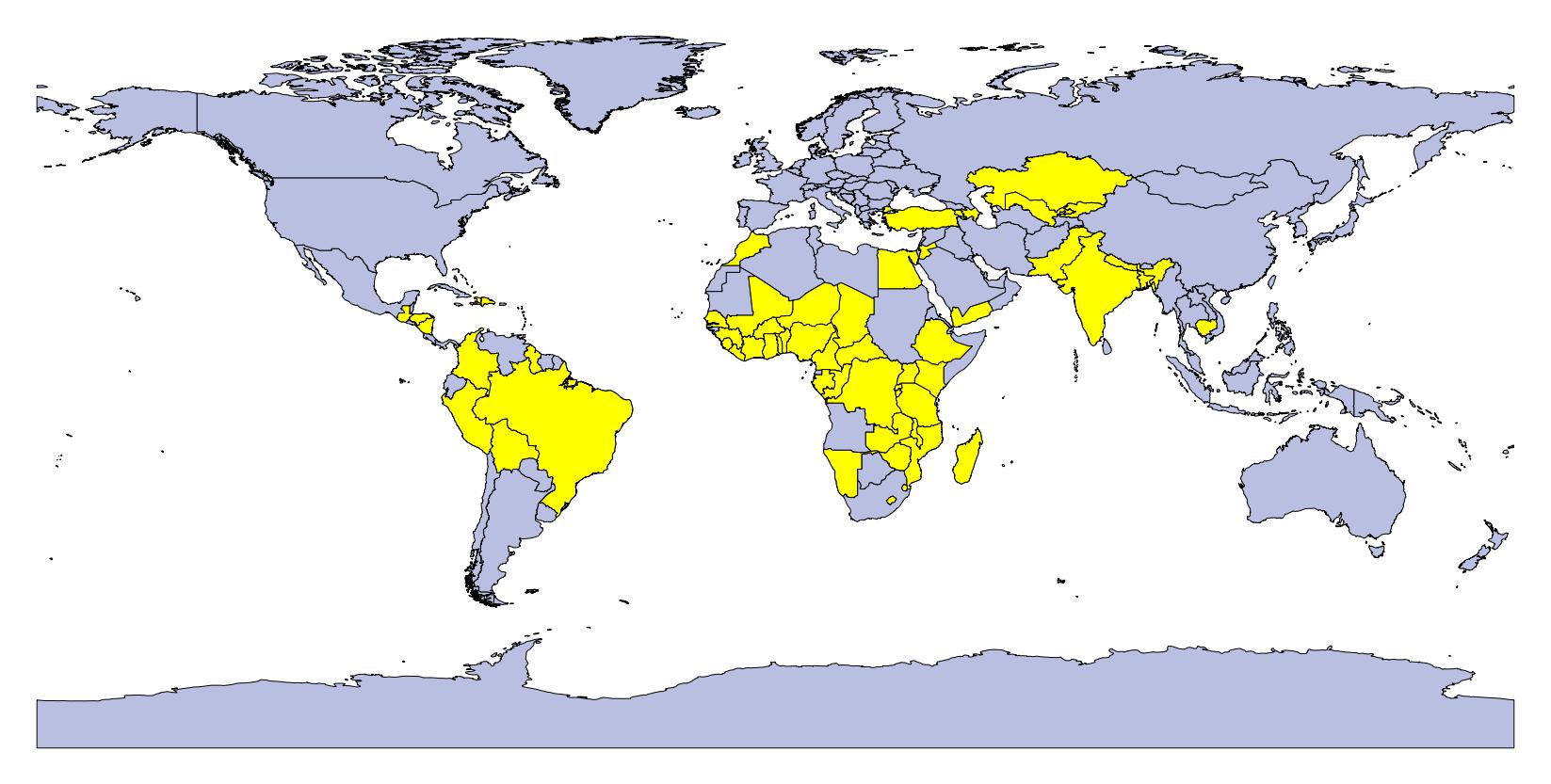


**Supporting Information Figure S5. Crude stunting prevalence for first born children by maternal age**

**
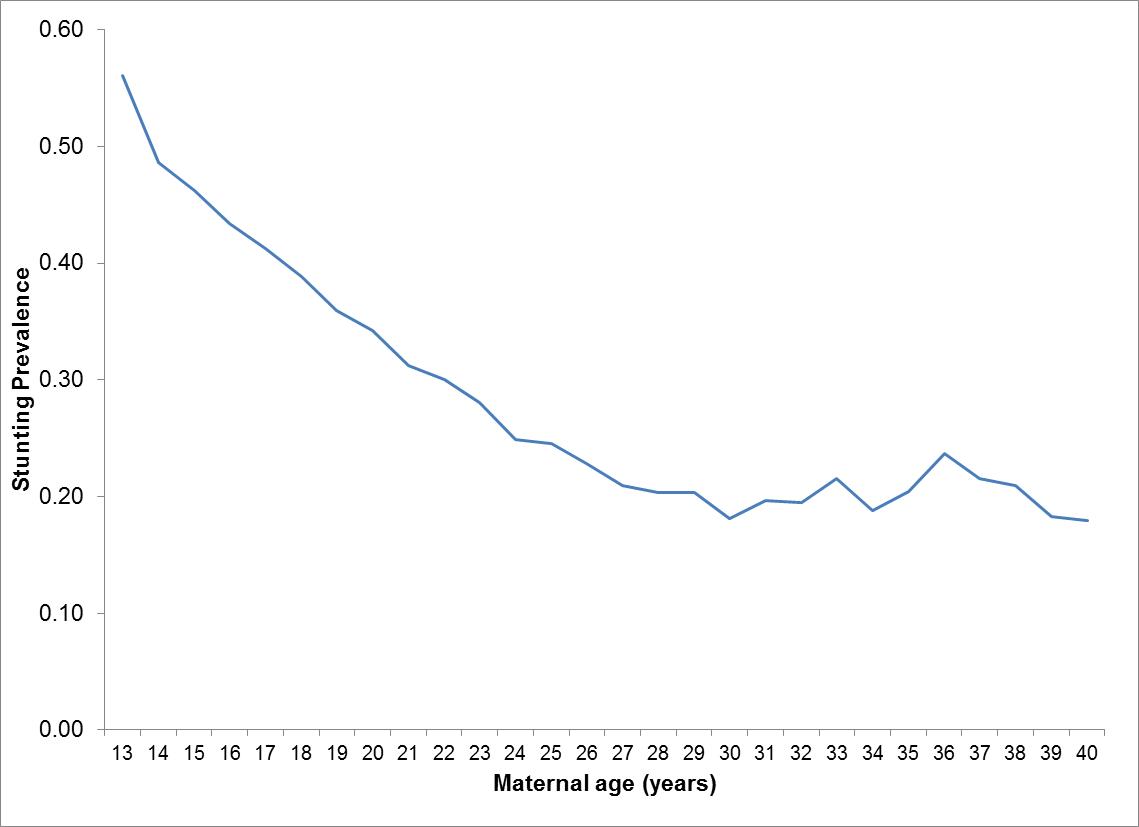
**

**Supporting Information Figure S6. Crude stunting prevalence for first born children by birth interval**

**
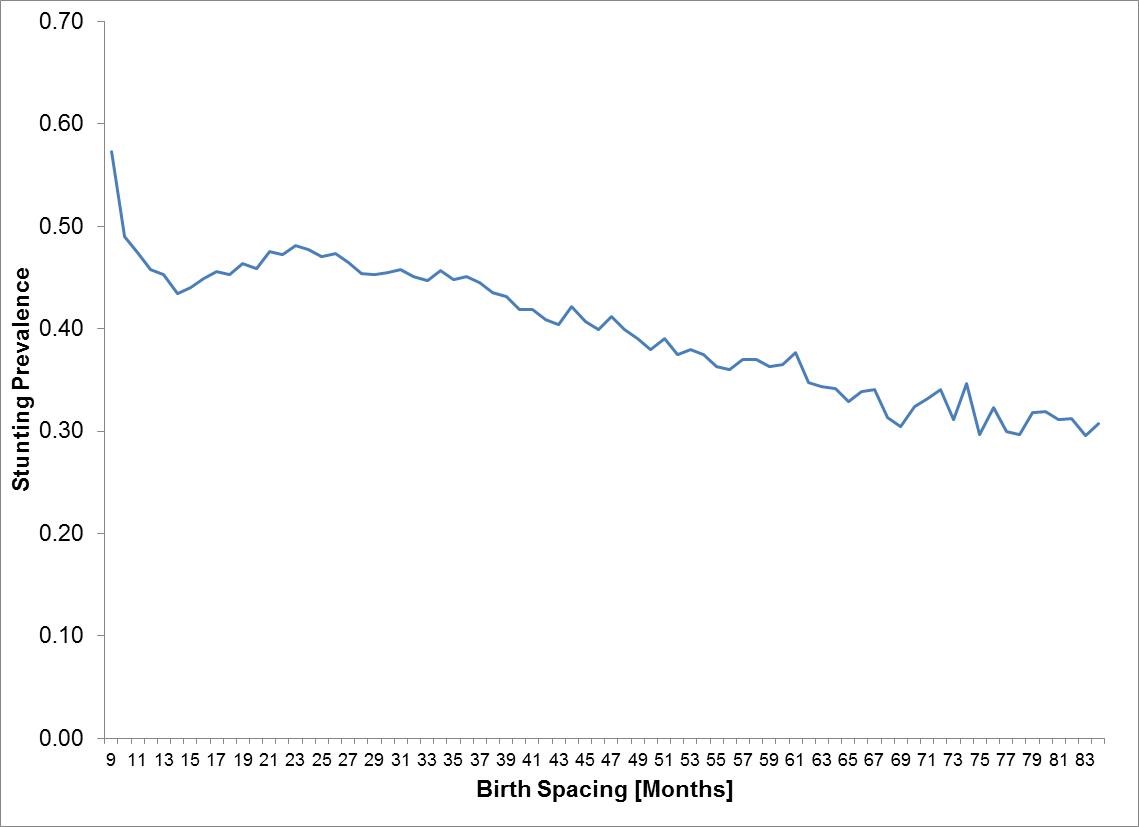
**
